# *Helicobacter pylori* CagA protein activates Akt and attenuates chemotherapeutics-induced apoptosis in gastric cancer cells

**DOI:** 10.18632/oncotarget.23050

**Published:** 2017-12-09

**Authors:** Keng-Hsueh Lan, Wei-Ping Lee, Yu-Shan Wang, Shi-Xian Liao, Keng-Hsin Lan

**Affiliations:** ^1^ Division of Radiation Oncology, Department of Oncology, National Taiwan University Hospital National Taiwan University Cancer Center, Taipei, Taiwan; ^2^ Department of Medical Research, Taipei Veterans General Hospital, Taipei, Taiwan; ^3^ Institute of Molecular Medicine and Bioengineering, National Chiao Tung University, Hsinchu, Taiwan; ^4^ Division of Gastroenterology and Hepatology, Department of Medicine, Taipei Veterans General Hospital, Taipei, Taiwan; ^5^ Department of Medicine, School of Medicine, National Yang-Ming University, Taipei, Taiwan; ^6^ Department and Institute of Pharmacology, National Yang-Ming University, Taipei, Taiwan; ^7^ Department and Institute of Biochemistry, National Yang-Ming University, Taipei, Taiwan

**Keywords:** Helicobacter pylori, CagA, Akt, chemotherapeutics

## Abstract

Infection with *cagA*-positive *Helicobacter pylori* is associated with a higher risk of gastric cancer. The *cagA* gene product, CagA, is translocated into gastric epithelial cells and perturbs host cellular biological functions. Etoposide, a topoisomerase II inhibitor widely used to couple DNA damage to apoptosis, is a common cytotoxic agent used for advanced gastric cancer. We investigate the effect of CagA on etoposide-induced apoptosis in gastric cancer cells to elucidate whether CagA play a role in gastric carcinogenesis via impairing DNA damage-dependent apoptosis. AGS cell lines stably expressing CagA isolated from *H. pylori* 26695 strain were established. In the presence of etoposide, viability of parental AGS cells was decreased in a time-and dose-dependent manner, whereas CagA-expressing AGS cells were less susceptible to etoposide induced cell-killing effect. Suppression of etoposide-induced apoptosis was shown in CagA-expressing but not in parental AGS cells by DNA fragmentation, cell cycle, and annexin-V assays. This inhibitory effect of etoposide-induced apoptosis conferred by CagA was also demonstrated in SCM1 and MKN45 gastric cancer cell lines, with two additional chemotherapeutics, 5-FU and cisplatin. The effect of Akt activation on inhibition of etoposide-induced cytotoxicity by CagA was also evaluated. CagA expression and etoposide administration activate Akt in a dose-dependent manner. Enhancement of etoposide cytotoxicity by a PI-3-kinase inhibitor, LY294002, was evident in parental but was attenuated in CagA-expressing AGS cells. CagA may activate Akt, either in the absence or presence of etoposide, potentially contributing to gastric carcinogenesis associated with *H. pylori* infection and therapeutic resistance by impairing DNA damage-dependent apoptosis.

## INTRODUCTION

*Helicobacter pylori* (*H. pylori*) is a Gram-negative bacterium infecting human gastric mucosa with worldwide prevalence. This bacterium contributes to the pathogenesis of chronic gastritis, peptic ulcers, gastric adenocarcinomas, and gastric mucosa–associated lymphoid tissue lymphomas [1]. Several studies have shown that cytokine production, apoptosis, and cellular proliferation are induced in gastric mucosa infected with *H. pylori*, and that these cellular responses play a crucial role in gastric inflammation, atrophy, hyperplasia, and carcinogenesis [2–6]. Increasing evidence suggests that *H. pylori* is causally linked to gastric cancer. A report summarized all available case-control studies and concluded that the relative risk for the development of gastric cancer was 3.8-fold higher in patients with *H. pylori* infection compared to those without infection. Additionally, this risk increased to 8.7-fold 15 years after the diagnosis of *H. pylori* infection [7]. In a meta-analysis of 42 cohort and case-control studies, Eslick *et al.* reported an association between *H. pylori* infection and gastric cancer with the overall odds ratio of 2.0 (95% CI 1.7–2.5) [8]. In animal studies, several groups have shown the development of adenocarcinoma in the antral or pyloric region of Mongolian gerbils infected with *H. pylori* [9–11]. Given the causal role of *H. pylori* infection in the development of gastric carcinoma, the World Health Organization has classified *H. pylori* as a class I carcinogen.

Several factors have been proposed to be possible virulence determinants of *H. pylori*, including the *cagA* gene, a marker gene for the *cag* pathogenicity island [12]. Among various *H. pylori* strains, *cagA*-positive *H. pylori* strains are more virulent than *cagA*-negative strains and are associated with gastric carcinoma. CagA is believed to evoke an increased inflammatory response through epithelial cell release of interleukin 8, leading to active inflammation [13]. The presence of this gene has been strongly linked to gastric mucosal cell damage [14]. After attachment of *cagA*^*+*^ *H. pylori* to gastric epithelial cells, CagA is directly injected into the cells via the bacterial type IV secretion system and undergoes tyrosine phosphorylation on specific EPIYA sequence repeats by Src family tyrosine kinases in the host cells [15–18]. The phosphorylated CagA specifically binds Src homology 2 domain-containing protein tyrosine phosphatase (SHP-2), activates the phosphatase activity, thereby inducing morphological transformation of cells [19]. These steps trigger downstream events leading to *H. pylori*-associated apoptosis and cellular proliferation. An international survey identified a strong association between CagA status and pepsinogen levels that are markers of gastric atrophy [20].

Etoposide induces DNA cleavage and prevents DNA strand reconnection, leading to DNA strand breaks. Cancer cells are more dependent on topoisomerase than healthy cells due to the higher proliferation rate. Thus, etoposide causes the errors in DNA synthesis and promotes apoptosis of cancer cells [21]. Etoposide is an important cancer chemotherapeutic agent with clinical activity against a broad range of human malignancies [22], including gastric cancer. Etoposide has been shown to activate phosphatidylinositol 3-kinase/Akt signaling pathway [23], which induces resistance of gastric cancer cells to chemotherapeutic agents [24, 25]. Administration of a small molecule inhibitor of the phosphatidylinositol 3-kinase (PI3K), LY294002, greatly potentiated apoptosis caused by etoposide [26].

CagA has been shown to promote gastric carcinogenesis directly by affecting host cell signaling pathways, or indirectly by inducing tissue damage and inflammation [27]. The purpose of this study is to evaluate whether the CagA of *H. pylori* is implicated in resistance of gastric cancer cells to etoposide-induced apoptosis and to explore the possible mechanism.

## RESULTS

### CagA-expressing AGS cells are less susceptible to etoposide-induced cytotoxicity

**Figure 1:**
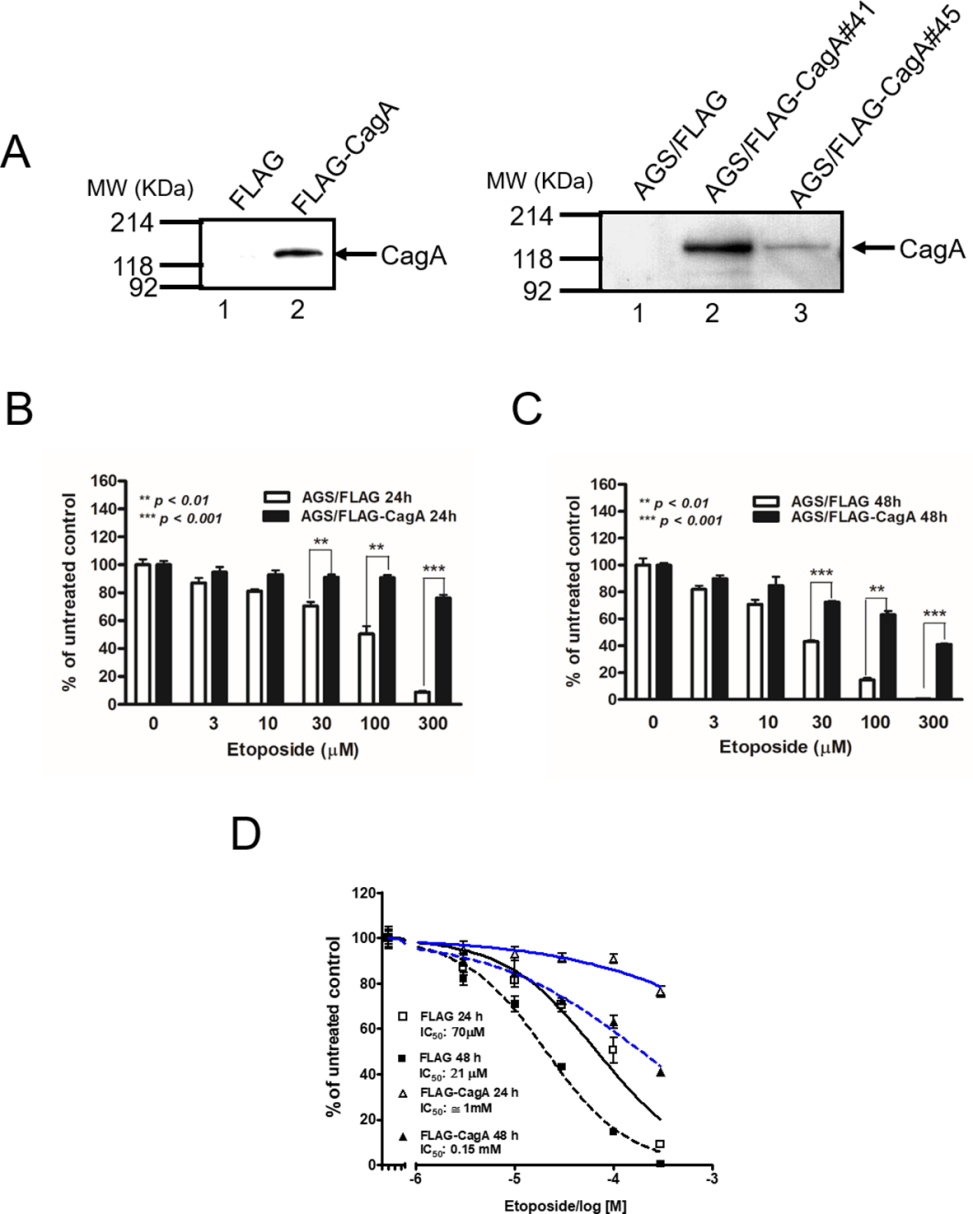
Effect of the CagA on cell viability after etoposide treatment (**A**) Specific CagA expression with the expected molecular weight 128 KDa was noted in AGS cells transiently transfected p3XFLAG-CagA but not in p3XFLAG control. CagA expression was also noted in two CagA-expressing AGS stable transfectants (*right panel*). (**B** and **C**) Control or stable CagA-expressing AGS cells (7 × 10^4^/well) were treated with etoposide at the concentrations indicated. After being cultured for 24 h (B) or 48 h (C), cells were subjected to MTT assay. Results are presented as mean ± SEM of three independent determinations. (**D**) Inhibition curves of the IC_50_ of AGS/FLAG and AGS/FLAG-CagA cells when exposed to etoposide for 24 h and 48 h were plotted using GraphPad Prism software.

Specific expression of CagA was noted in transiently transfected AGS cells and two stable transfectants (Figure 1A). To determine the effect of CagA expression on etoposide-induced cytotoxicity, AGS/FLAG and AGS/FLAG-CagA cells were treated with various concentrations of etoposide (0–300 μM) for 24 to 48 h. The AGS/FLAG cells were susceptible to etoposide in a dose- and time-dependent manners while the AGS/FLAG-CagA cells demonstrated attenuated cytotoxicity (Figure 1B and 1C). While the concentration of etoposide causing 50% of the cell death (IC_50_) after 48 h of treatment is 21 µM for AGS/FLAG cells, the IC_50_ was increased to 150 µM for AGS/FLAG-CagA cells (Figure 1D). The difference was even more evident after drug exposure for 24 h, with the IC_50_ being 14 folds higher in CagA-expressing than parental AGS cells (1 mM *vs* 70 μM) (Figure 1D).

### CagA prevents etoposide-induced DNA fragmentation and PARP cleavage

**Figure 2:**
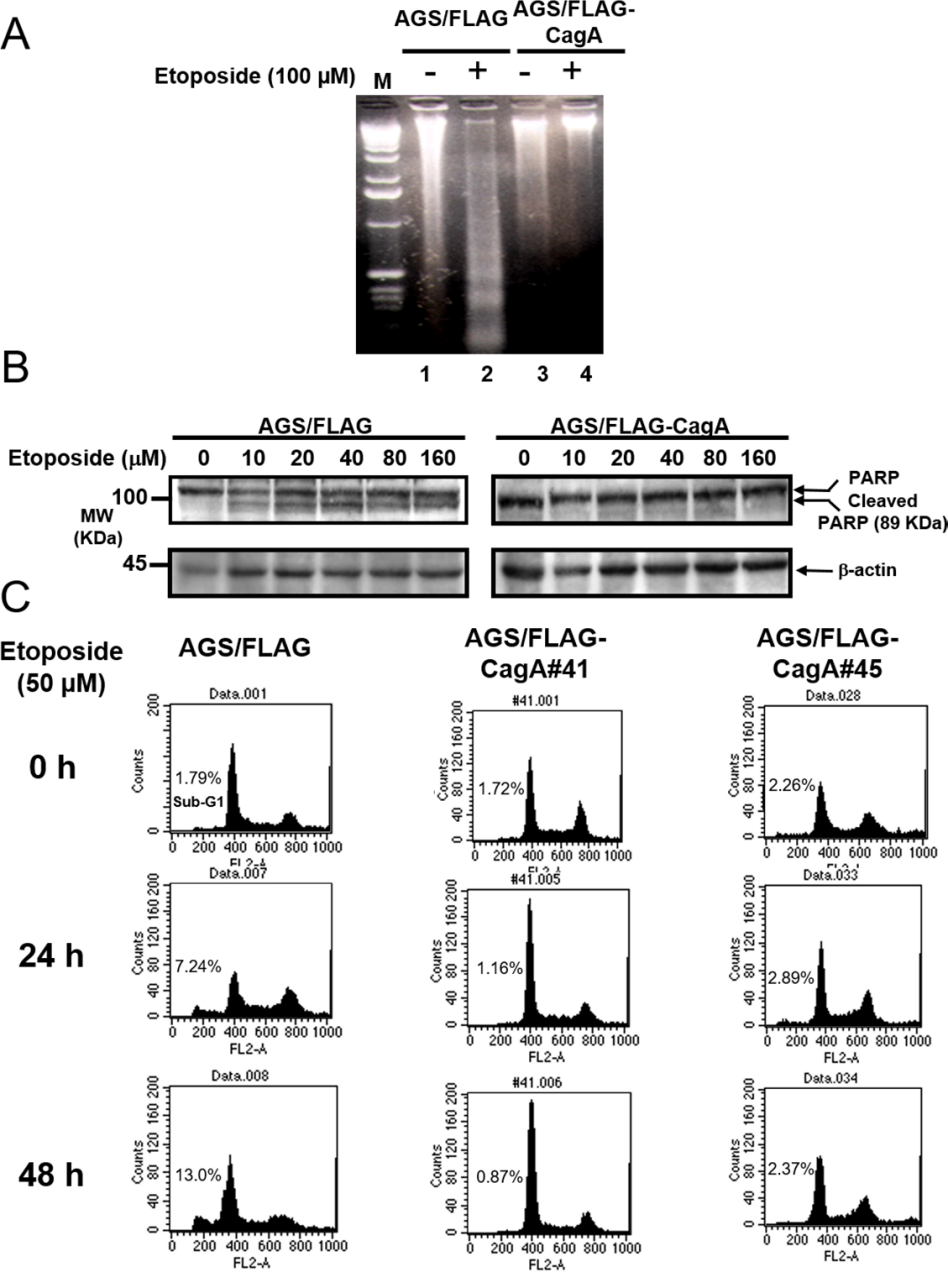
CagA expression resists etoposide-induced apoptosis (**A**) DNA ladder formation assay. Control AGS cells untreated (*lane 1*) or treated with 100 µM of etoposide (*lane 2*) and AGS/FLAG-CagA#41 cells untreated (*lane 3*) or treated with 100 µM of etoposide (*lane 4*) were subjected to 1.5% agarose gel electrophoresis, followed by staining with ethidium bromide.. Etoposide enhanced levels of DNA ladder formation in AGS cells (*lane 2*) but not in AGS/FLAG-CagA#41 cells (*lane 4*). Representative data from one of two similar experiments are presented. (**B**) Serum-starved AGS/FLAG or AGS/FLAG-CagA#41 cells were treated with etoposide at the concentrations indicated for 6 h. The cell lysates (50 µg) were analyzed for PARP and β-actin by Western blotting. Cleaved PARP, MW: 89 KDa. (**C**) Control and two stable CagA-expressing AGS cell lines (AGS/FLAG-CagA #41, and #45) were treated with 50 µM of etoposide for the time indicated. Apoptosis was quantitated by propidium iodide staining and flow cytometry. Representative histograms were shown to demonstrate the appearance of a sub-G_1_ peak in etoposide treated cells.

Degradation of chromosomal DNA into oligonucleosomal fragments represented by multiples of 180–200 bp DNA on agarose gel electrophoresis is one of the hallmarks of apoptosis. DNA ladder formation was evident in AGS/FLAG cells after etoposide treatment for 48 h (Figure 2A; *lane 2*) but not in untreated AGS/FLAG cells (*lane 1*) or AGS/FLAG-CagA cells untreated (*lane 3*) or treated (*lane 4*) with etoposide. These results indicate that etoposide induced apoptotic cell death in AGS cells, whereas CagA expression suppresses the apoptotic effect of etoposide.

PARP is a nuclear DNA-binding protein that detects and binds to DNA strand breaks. Full-length PARP is a 116 KDa stress response protein that repairs damaged DNA. Proteolytic cleavage of PARP into 89 KDa and 24 KDa fragments by caspases is a well-established hallmark and an early indicator of apoptosis. To evaluate the cytoprotective actions of CagA, the levels of cleaved PARP in parental or CagA-expressing AGS cells were examined following treatment of various concentrations of etoposide (0–160 μM) for 6 h. PAPR cleavage was evident by etoposide treatment in AGS/FLAG (Figure 2B; left panel) but the effect was attenuated in AGS/FLAG-CagA cells (right panel).

### Etoposide-induced apoptosis was blocked in stable CagA-expressing AGS cells

To further evaluate the impact of CagA expression upon etoposide-induced apoptosis, two AGS cell lines stably transfected with CagA (AGS/FLAG-CagA #41, and #45) were treated with 50 μM etoposide for the time indicated. Cells were stained with propidium iodide, and apoptosis was scored by the appearance of a sub-G_1_ peak, a characteristic of apoptosis. Before the treatment of etoposide, there was no significant difference in the sub-G_1_ peaks of the control AGS/FLAG and AGS/p3XFLAG-CagA cells. While 7.24% and 12.99% of apoptotic cells appeared in AGS/FLAG cells after 24 and 48 h treatments, respectively (Figure 2C; left panel), both AGS/p3XFLAG-CagA stable cells showed less than 3% of apoptotic cells at the corresponding time points (middle and right panel).

### CagA-expressing gastric cancer cell lines attenuate apoptosis induced by chemotherapy drugs

**Figure 3:**
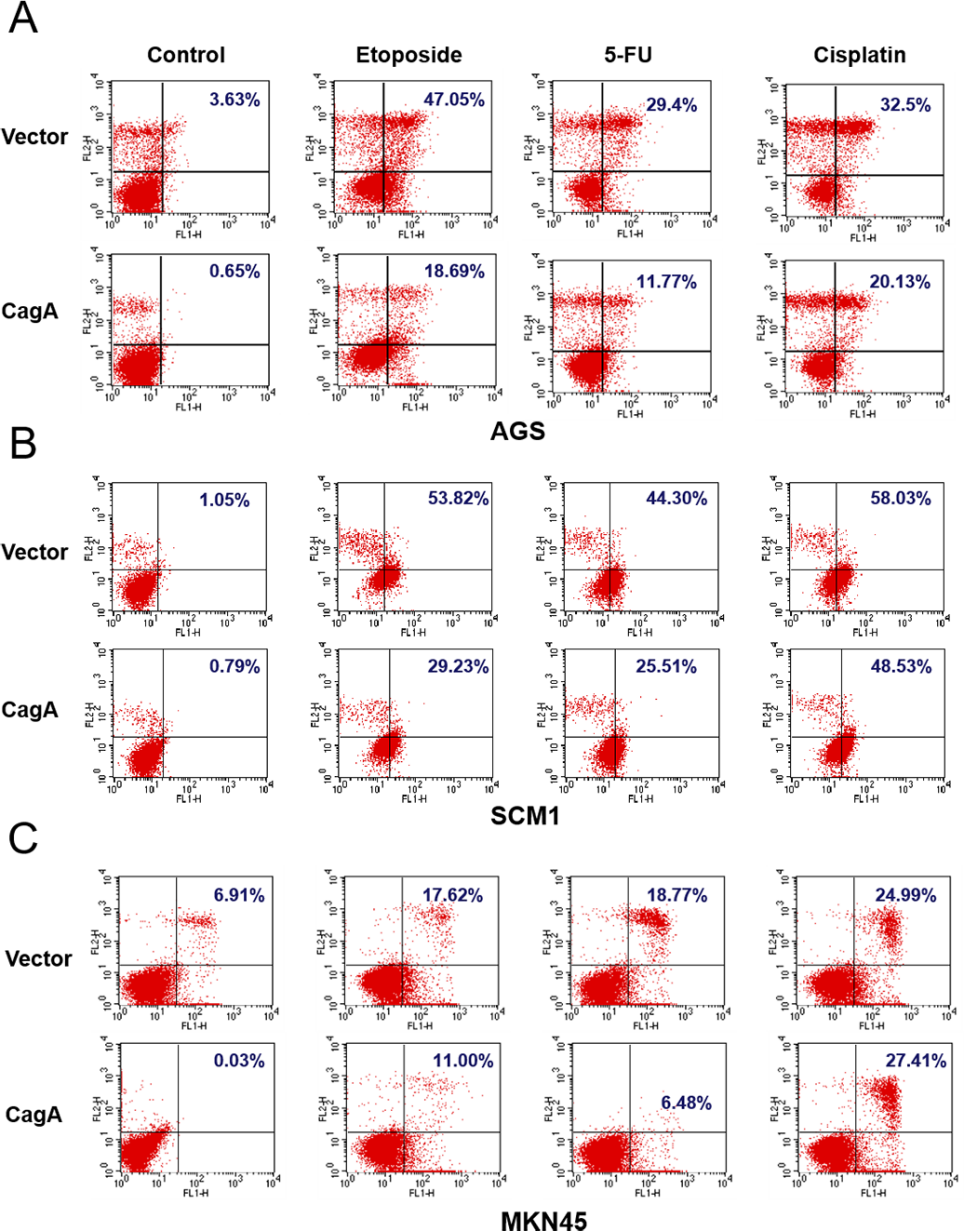
CagA expression attenuates the cell death induced by chemotherapeutic drugs in three gastric cancer cell lines Annexin V/ PI staining of gastric cancer cells, (**A**) AGS (**B**) SCM1 (**C**) MKN45, transfected with vector or CagA-expressing plasmids followed by treatment of three different chemotherapeutics, etoposide, cisplatin or 5-FU, at a concentration of 33 μM, for 24 hours.

To further confirm the apoptosis blockage phenomenon induced by CagA on gastric cancer cells, two additional gastric cancer cell lines and another two chemotherapeutic agents, 5-FU and cisplatin, were used to evaluate the phenomenon. CagA transfected AGS, SCM1 and MKN45 cell lines were treated with etoposide, 5-FU and cisplatin for the 24 h and analyzed by annexin-V assay. As shown in Figure 3A, apoptotic cell death was attenuated when CagA-expressing cells were treated with etoposide, 5-FU or cisplatin from 47.05% of vector control to 18.69% in etoposide, from 29.4% of vector control to 11.77% in 5-FU and from 32.5% to 20.13% in cisplatin. The expression of CagA weakened the execution stages of apoptosis in another gastric cancer cell line (SCM1), also treated with etoposide, 5-FU or cisplatin (Figure 3B). Vector control of SCM1 showed 53.82%, 44.30% and 58.03% of apoptosis on treatment of etoposide, 5-FU and cisplatin, respectively, whereas CagA-expressing SCM1 cells attenuated apoptosis to 29.23%, 25.51% and 48.53% (Figure 3B). To validate the observations that the expression of CagA affects cell sensitivity to the studied drugs, we assessed the outcome of etoposide, 5-FU and cisplatin on another gastric cell line, MKN45. As shown in Figure 3C, CagA-expressing MKN45 cells blocked etoposide-induced apoptosis from 17.62% of vector control to 11.00%, 5-FU-induced apoptosis from 18.77% of vector control to 6.48%. However, the CagA-expressing MKN45 cells treated with cisplatin did not result in changes in the level of apoptosis in comparison with MKN45 vector control cells.

### Activation of Akt by CagA in gastric cancer cells

**Figure 4:**
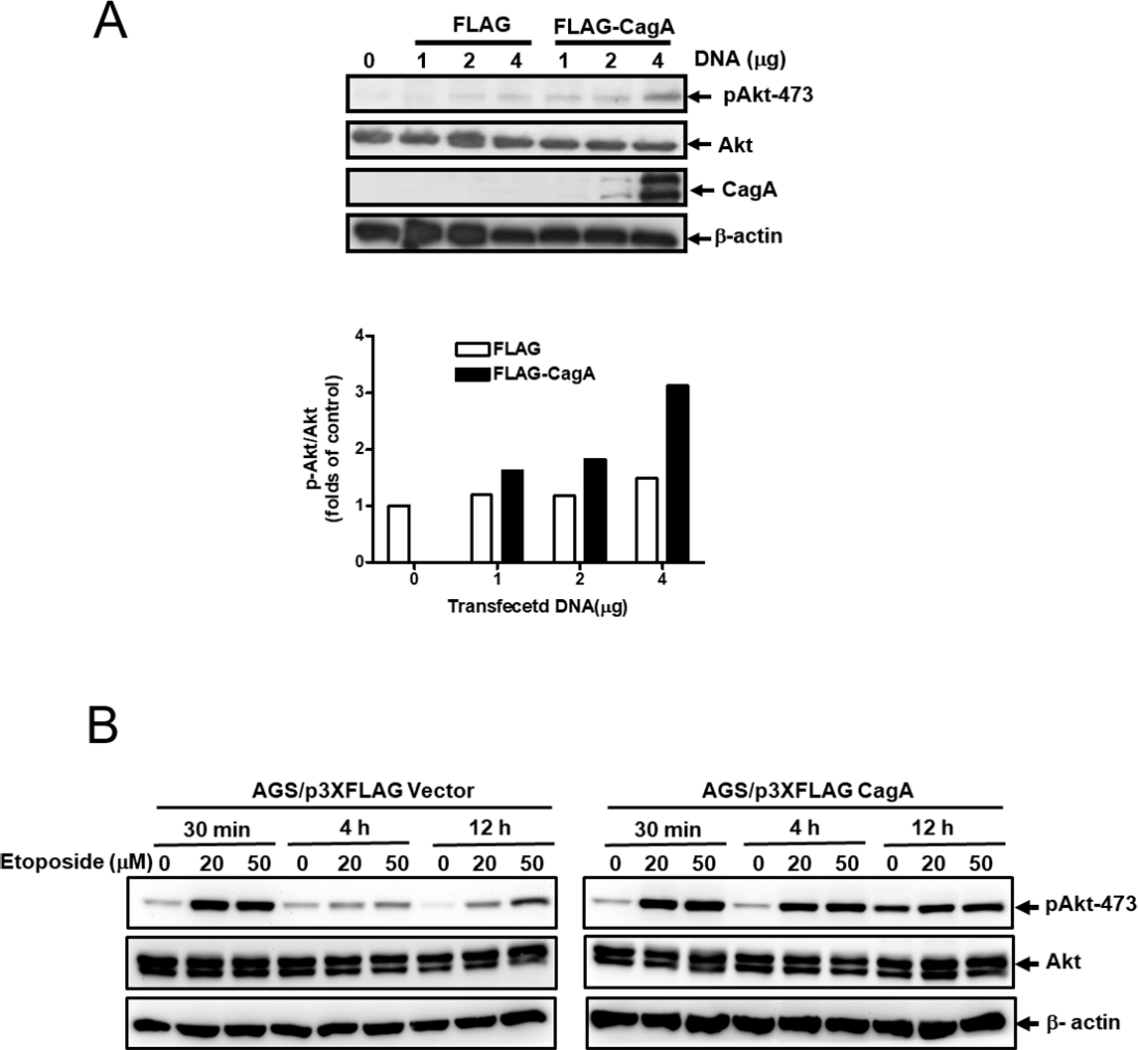
CagA expression activates Akt in gastric cancer cell (**A**) AGS cells were transfected with p3XFLAG vector or p3XFLAG-CagA plasmid as indicated amounts. Cell lysates were analyzed by Western blot with anti-phospho-Akt (Ser 473), total Akt, CagA and β-actin antibodies. The relative expression levels of phospho-Akt (Ser 473) to total Akt were semi-quantified by Image J software. (**B**) AGS/FLAG or AGS/FLAG-CagA stable cells were left untreated or treated with etoposide (20 or 50 µM) for 30 min to 12 h, and cell lysates were analyzed by Western blot with anti-phospho-Akt (Ser 473), total Akt, and β-actin antibodies. The relative expression levels of phospho-Akt (Ser 473) to total Akt were semi-quantified by Image J software.

To investigate the relationship of CagA expression and Akt activation in gastric cancer cells, we examined the p473-Akt level in AGS after transfection with various amount of p3XFLAG vector or p3XFLAG-CagA plasmid (0–4 µg). Total Akt levels were not influenced by the nature or amount of transfected DNA (Figure 4A, first panel). However, the phosphorylated Akt (p473-Akt) levels increased in response to CagA expression in a dose dependent manner (Figure 4A, second panel). The relative p473-Akt level was most evident after transfection with 4 μg of CagA-expressing plasmid, up to three folds as compared with control (Figure 4A, graph).

To elucidate the role of activation of Akt in resistance to etoposide-induced apoptosis by CagA, AGS/FLAG and AGS/FLAG-CagA cells were treated with etoposide for 30 min to 12 h to determine the effect of CagA on Akt activation in response to short- and long-time etoposide treatments. Consistent with previous results, activation of Akt was evident following etoposide treatment for 30 min in both AGS/FLAG and AGS/FLAG-CagA cells (Figure 4B). While Akt activation decayed in AGS/FLAG cells, CagA maintained persistent p473-Akt expression after long-time etoposide treatment (4 h and 12 h) (Figure 4B).

### Enhancement of etoposide cytotoxicity by PI-3-kinase inhibitor LY294002 was attenuated in CagA-expressing gastric cancer cells

**Figure 5:**
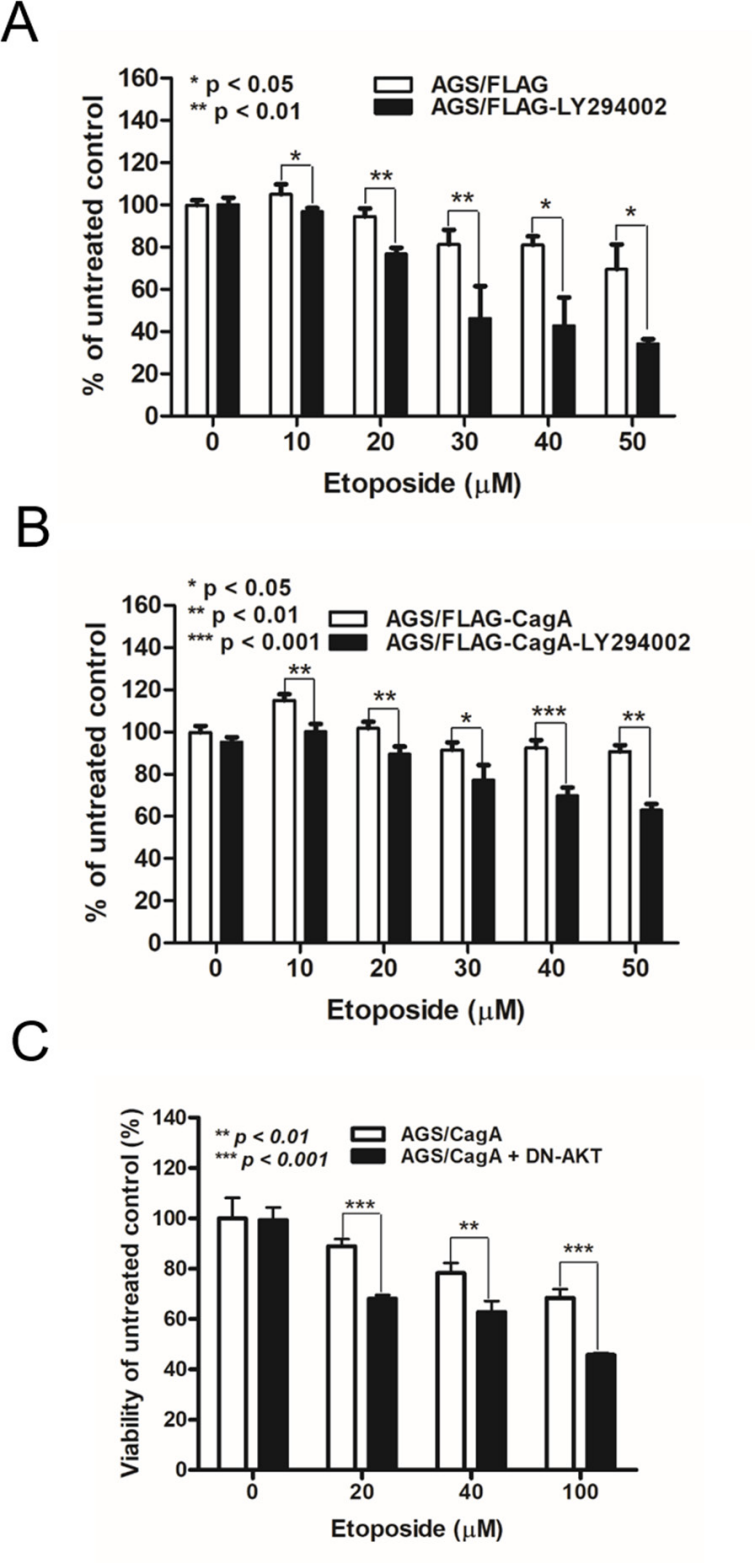
LY294002 and dominant-negative Akt mutant alter the sensitivity of CagA-expressing AGS cells to etoposide (**A**) Control FLAG- or (**B**) FLAG-CagA-expressing stable AGS cells were plated at 5 × 10^4^/well and placed in serum-free medium for 24 h before being treated with etoposide at various concentrations (0–50 µM) and LY294002 for 48 h. MTT assay was performed to determine the cell viability. (**C**) AGS/FLAG-CagA stable cells were transiently transfected with DN-Akt, and cell viability in response to etoposide treatment was assessed by MTT assay.

To scrutinize whether CagA confers the resistance against chemotherapeutics via Akt activation, we examined the effect of PI-3-kinase inhibitors LY294002 on cell viability of AGS/FLAG and AGS/FLAG-CagA cells in the presence or absence of etoposide. The addition of LY294002 significantly enhanced cytotoxicity of etoposide in AGS/FLAG cells (Figure 5A), and to a lesser extent in the CagA-overexpressing AGS/FLAG-CagA cells (Figure 5B). To confirm that the effect of LY294002 on etoposide-induced cytotoxicity were attributable to inhibition of Akt, AGS/FLAG-CagA cells transfected with dominant negative Akt (DN-Akt) were assessed for cell viability after etoposide treatment. While AGS/FLAG-CagA cells had higher Akt activity (Figure 4A) and showed cytoprotection against etoposide-induced apoptosis, transfection of DN-Akt augmented etoposide-induced cytotoxicity (Figure 5C). This result further confirmed that Akt signaling pathway is selectively involved in etoposide-induced cytotoxicity in AGS cells.

## DISCUSSION

Adenocarcinoma of the stomach is the second most common cancer causing mortality in the world. *Helicobacter pylori* (*H. pylori*) is a well known carcinogen of gastric cancer. It has been shown that *H. pylori* strains expressing the cytotoxin-associated protein (CagA) associate high prevalence with gastric cancer as compared with CagA-negative strains [28]. CagA is secreted by *H. pylori* and translocated into gastric epithelial cells by type IV secretion system [18, 29]. CagA interacts with SHP-2 inducing a growth factor-like response in gastric epithelial cells [20]. Moreover, CagA, when co-expressed with HspB, induces cell proliferation in AGS gastric epithelial cells [30]. As a result, CagA has been suggested as a potential bacterial oncoprotein in gastric carcinogenesis [31].

Etoposide binds to DNA and topoisomerase II, by which the drug induces apoptosis in AGS cells. Cag A activates Akt that suppresses apoptosis by phosphorylating, and therefore inhibiting, pro-apoptotic proteins, such as BAD [32], ASK1 [33] and caspase 9 [34]. Akt also suppresses apoptosis by promoting the degradation of IκB, which leads to the activation of NF-κB [35] and to the suppression of apoptosis via the transcription of anti-apoptotic genes, such as Bcl-2 [36], Bcl-XL [37], and IAP [38]. Akt may also directly stimulate DNA repair exerted by etoposide. Following DNA damage, Akt binds to DNA-PK, phosphorylates it and regulates the accumulation of DNA-PK at damaged sites to assist DNA double strand break re-joining by non-homologous end joining (NHEJ) [39]. Akt signaling is activated in gastric cancer, which has been implicated in tumorigenesis of gastric cancer [40], influencing the chemoresistance of gastric cancers [41, 42], and correlating with the grade of malignancy in human gastric adenocarcinomas [43]. Besides, infection of CagA-positive *H. pylori* strains activates Akt in gastric epithelial cell lines, including AGS [44–47], MKN45 [45], MKN28 [45], and nontransformed epithelial cell line, MCF-10A [48], which attenuates cell apoptosis and promotes cell survival. Akt activation is a common observation in response to chemotherapy, including etoposide, suggesting an important role in inducing resistance to apoptosis in breast [26], small cell lung [49] and gastric cancer cells [24]. Our results showed that etoposide increased Akt phosphorylation as well as decreased cell viability through induction of apoptosis in AGS cells. However, this etoposide-induced apoptosis was much attenuated in CagA-expressing AGS gastric cancer cells, which may be attributable to further Akt activation by CagA expression.

We continued to test the chemoresistance extent conferred by CagA in AGS, and two additional gastric cancer cell lines, SCM1, and MKN45 treated with multiple chemotherapeutics including etoposide, 5-FU, or cisplatin (Figure 3). The results showed that in all three gastric cancer cell lines, CagA mediated cytoprotection against etoposide and 5-FU. CagA expression also exert some cytoprotection against cisplatin in AGS cells, but not in the other two gastric cancer cell lines (Figure 3, Cisplatin column). It is therefore intriguing to speculate that the extent of CagA-mediated chemoresistance may involve the interplay between the tumor cell factors and the action of the antitumor compounds.

Our data in Figure 4 corroborate the correlation of CagA expression and Akt activation. The phosphorylated Akt (P-Ser473-Akt) levels were increased with the increasing amounts of transfected CagA DNA (Figure 4A). Interestingly, the enhanced Akt activation with increasing etoposide concentration is much more significant in the presence of CagA expression (Figure 4B). These results indicate that CagA mediated chemoresistance via Akt activation.

Administration of a potent PI3K inhibitor, LY294002 or transient transfection of a DN-Akt mutant inhibits Akt phosphorylation and greatly potentiates apoptosis caused by etoposide [24, 26]. In our study, etoposide-induced apoptosis was potentiated by LY294002 in control but to a lesser extent in CagA-expressing AGS cells, suggesting CagA may attenuate etoposide-mediated apoptosis via PI3K/Akt signaling pathway. To further elucidate the role of Akt in attenuation of etoposide-induced apoptosis by CagA, DN-Akt was transfected into CagA-expressing AGS cells followed by etoposide treatment. In agreement with our hypothesis, DN-Akt potentiated the etoposide-induced apoptosis even in CagA-expressing AGS cells, indicating that CagA suppresses etoposide-mediated apoptosis by Akt signaling pathway.

Many oncoproteins, survival factors, and growth factors have evolved various mechanisms for Akt activation. Given that Akt activation often correlates with cellular response to carcinogen [25], our data may have an important implication for cellular transformation induced by CagA-positive *H. pylori*. In light of the CagA-induced Akt activation, CagA may be a potential bacterial oncoprotein in gastric carcinogenesis. Furthermore, CagA potentiates Akt activation induced by etoposide by which it might lead to resistance of gastric cancer to standard chemotherapy. Therefore, it is plausible to determine the effect of CagA-positive *H. pylori* infection on treatment efficacy of chemotherapeutics in gastric cancer patients.

Taken together, this is the first study showing that CagA of *H. pylori* potentiates Akt activation and attenuates the etoposide-induced apoptosis in gastric cancer cells. CagA activates multiple anti-apoptotic signaling pathways of host cells, the PI3K/Akt activation of which is responsible for *H. pylori*-induced tumorigenesis of gastric cancer [40]. This CagA-dependent mechanism of etoposide resistance in gastric cancer cells may help develop state-of-the-art chemotherapeutic regimens. Since CagA activates oncogenic signaling pathway, pathological detection of CagA in gastric cancer surgical specimens may also have prognostic as well as therapeutic significance in clinical practices.

## MATERIALS AND METHODS

### Cell lines and reagent

Human gastric cancer cells, AGS (ATCC CRL-1739) and SCM1 (ATCC CRL-5822), were obtained from American Type Culture Collection. MKN45 cell line (RCB1001) was obtained from RIKEN BioResource center. The cells were maintained in RPMI-1640 medium supplemented with 10% fetal calf serum, 50 µg/ml penicillin-streptomycin and 2 mM L-glutamine at 37°C in a 5% CO_2_ atmosphere. Etoposide, cisplatin, 5-Fluorouracil (5-FU), and LY294002 were purchased from Sigma (Saint Louis, Missouri, USA).

### Plasmids

The full-length *cagA* gene was amplified from *H. pylori* strain 26695 by polymerase chain reaction (PCR) with specific primers containing unique *Kpn*I sites (5′-GG*GGTACC*CACTAACGAAACTATTGATCAAACAAG-3′; 5′-GG*GGTACC*CTTAAGATTTTTGGAAACCACCTTTTG-3′). The PCR product was cloned into the *KpnI* site of the expression vector p3XFLAG-Myc-CMV™-26 (Sigma, Saint Louis, Missouri, USA) to generate p3XFLAG-CagA where upstream of full-length *cagA* gene is tagged with three adjacent FLAG epitopes (Asp-Tyr-Lys-Xaa-Xaa-Asp). Akt was tagged with HA (YPYDVPDYA) at its C-terminal end to generate Akt-HA (wt). Dominant-negative Akt (DN-Akt) was constructed by site-directed mutagenesis at the ATP binding site (K179M) from Akt-HA to express a kinase-dead Akt. All cloned plasmids were purified using Endofree plasmid kit (Qiagen, Hilden, Germany).

### Antibodies

For Western blot analysis, rabbit polyclonal antibodies against phospho-Akt (Ser473, catalog no. 9271, 1:1,000 dilution) and nonphosphorylated total Akt (catalog no. 9272, 1:500 dilution) were purchased from Cell Signaling Technology (Beverly, MA). Anti-FLAG M2 and anti-β-actin antibodies were obtained from Sigma. Antibodies against poly(ADP-ribose) polymerase (PARP) were purchased from Santa Cruz Biotechnology, Inc. (Santa Cruz, CA).

### Stable CagA expression gastric cancer cell lines

For selection of FLAG-CagA or FLAG stable transfectants, AGS, SCM1 and MKN45 cells were transfected with p3XFLAG-CagA or p3XFLAG vector plasmid and selected by resistance to G418 (Gibco-BRL) at the concentration of 400 µg/mL. The protein expression was confirmed by Western blots.

### Immunoblot analysis

AGS cells (4 × 10^5^) were plated onto a 6-well tissue culture plate 24 hours before transfection with p3XFLAG-CagA or p3XFLAG plasmid using FuGENE 6 transfection reagent (Boehringer Mannheim, Mannheim, Germany). After 48 h, cells were suspended in 50 mmol/L Tris-HCl (pH 7.4) buffer containing 1 mmol/L EGTA, 2 mmol/L dithiothreitol, 25 mmol/L sodium beta-glycerophosphate, 0.1 mmol/L phenylmethylsulfonyl fluoride, and 10 µg/mL aprotinin. An equal amount of protein extracts was fractionated by sodium dodecyl sulfate-polyacrylamide gel electrophoresis (SDS-PAGE) and transferred to a polyvinylidene difluoride membrane (Amersham Pharmacia Biotech, Buckinghamshire, England). The membrane was probed with anti-FLAG M2 antibodiy. AGS/FLAG and AGS/FLAG-CagA stable cells were treated with varying concentrations of etoposide (20 or 50 μM) for 30 min to 12 h, and the cell lysates were subjected to immunoblotting with anti-P-Ser473-Akt antibody followed by reprobing with anti-Akt or anti-β-actin antibodies.

### *In vitro* cell viability analysis

The *in vitro* cell viability of the cell lines were assessed by MTT (3-[4,5-dimethylthiazol-2-yl]2,5-diphenylterazolium bromide) assay. Control or stable CagA-expressing AGS cells (5 × 10^4^/well) were plated in 96-well and treated with etoposide at various concentrations (0–300 µM) for 24 to 48 h. One hundred microliters of MTT reagent (0.5 mg/mL) was then added to each well. Cells were cultured for an additional 4 h and absorbance at 570 nm was measured.

### DNA fragmentation analysis

Approximately 2 × 10^6^ control or stable CagA-expressing AGS cells plated in 10 cm-diameter dishes were untreated or treated with 100 μM of etoposide for 48 h. Cells were collected, and the cell pellets were mixed with 20 mL of lysis buffer (100 mM Tris-Cl, 20 mM EDTA, pH 8.0, 0.8% SDS). The lysate was then digested with 10 mL of RNase (100 µg/ml) at 37°C for 2 h and subsequently incubated with 10 mL of proteinase K (20 mg/ml) at 50°C for 90 min. After extraction with phenol and chloroform, the DNA was then precipitated with ethanol and dissolved in TE buffer. An equal amount of DNA from each sample was analyzed on a 1.5% agarose gel electrophoresis.

### Cleavage of PARP

AGS/FLAG or AGS/FLAG-CagA#41 cells were treated with serum starvation followed by etoposide treatment of various concentrations of etoposide (0–160 µM) for 6 h. The cell lysates (50 µg) were analyzed for PARP and β-actin by Western blotting.

### Cell cycle assay

Control (AGS/FLAG) or two stable CagA-expressing AGS cell lines (AGS/FLAG-CagA#41 and AGS/FLAG-CagA#45) plated in 10 cm-diameter dishes were treated with 50 µM of etoposide for 0–48 h. Etoposide-treated cells were trypsinized, washed with PBS and then fixed in 1 mL 70% ice-cold methanol. The cells were resuspended in 1 ml 50 μg/ml propidium iodide staining solution containing 0.1% Triton X-100 and 2 mg/ml RNase A at room temperature for 1 hour in the dark. Subsequently, the nuclei were subjected to DNA fragmentation analysis in a FACScalibur (Becton Dickinson, San Jose, CA) and Cell Quest Pro software (Becton Dickinson, Mountain View, CA). Based on propidium iodide staining, nuclei in the sub-G_1_ marker window represent apoptotic cells.

### Annexin-V apoptosis assay

Control or stable CagA-expressing AGS, SCM1 and MKN45 cells (5 × 10^5^/well) were plated in 6-well and treated with etoposide (33 µM), 5-FU (33 µM) or Cisplatin (33 µM) for 24 h, trypsinized, and washed twice with PBS. Apoptosis was confirmed using an Annexin V Apoptosis Kit (BD Pharmingen) according to the manufacturer’s instructions. Briefly, cells were washed 3 times with PBS; then, cells were analyzed immediately for apoptosis using Annexin V/PI staining. Washed cells were supplemented with 1% BSA and then stained directly with 10 µL of PI and 2.5 µL Annexin V-FITC after the addition of 222.5 µL of binding buffer. Immediately after 10 min of incubation in the dark on ice, the cells were analyzed by flow cytometry. The percentage of positive cells was determined by using a FACSCalibur cytometer and Cell Quest Pro software.

### Transient transfections

AGS/FLAG-CagA or AGS/FLAG cells (1 × 10^5^/well) plated in 12-well dishes were transfected with 2 µg of DN-Akt-HA plasmid in triplicate using the FuGENE 6. After 24 h, the cells were treated with increasing concentrations of etoposide for 24 h. Cell viability was determined using MTT assay.

### Data analysis

Data are given as mean ± standard deviation of at least two independent experiments. Data fitting and statistical analyses were computed using the GraphPad Prism program version 5.0 (Graph Pad Software, San Diego, CA).
